# Shared Mutations in Emerging SARS-CoV-2 Circulating Variants May Lead to Reverse Transcription-PCR (RT-PCR)-Based Misidentification of B.1.351 and P.1 Variants of Concern

**DOI:** 10.1128/Spectrum.00816-21

**Published:** 2021-10-13

**Authors:** Carmen Losada, Carla Rico-Luna, Álvaro Otero-Sobrino, Andrea Molero-Salinas, Sergio Buenestado-Serrano, Ana Candela, Laura Pérez-Lago, Patricia Muñoz, Pilar Catalán, Darío García de Viedma

**Affiliations:** a Gregorio Marañón General University Hospital, Madrid, Spain; b Instituto de Investigación Sanitaria Gregorio Marañón (IiSGM), Madrid, Spain; c CIBER Enfermedades Respiratorias (CIBERES), Madrid, Spain; d Complutense University of Madrid, Madrid, Spain; University of Cincinnati

**Keywords:** SARS-CoV-2, VOC, reverse transcriptase PCR, whole-genome sequencing, COVID-19, RT-PCR, WGS

## Abstract

Reverse transcription-PCRs (RT-PCRs) targeting SARS-CoV-2 variant of concern (VOC) mutations have been developed to simplify their tracking. We evaluated an assay targeting E484K/N501Y to identify B.1.351/P1. Whole-genome sequencing (WGS) confirmed only 72 (59.02%) of 122 consecutive RT-PCR P.1/B.1.351 candidates. Prescreening RT-PCRs must target a wider set of mutations, updated from WGS data from emerging variants.

## OBSERVATION

The recent emergence and successful spread of SARS-CoV-2 variants of concern (VOCs) have triggered alarm (1). Definitive identification of these variants requires whole-genome sequencing (WGS) to identify the constellation of variant-specific mutations (2). In the search for a higher throughput, lower costs, and faster availability of data, alternative reverse transcription-PCR (RT-PCR)-based approaches have been proposed to prescreen these variants. For the first VOC triggering a global alarm, namely, B.1.1.7, an RT-PCR approach (Thermo Fisher TaqPath RT-PCR) took advantage of spike gene (S-gene) impaired detection, caused by the 69/70 deletion, which is a B.1.1.7 genetic marker (3). This RT-PCR demonstrated its usefulness to simplify and accelerate the B.1.1.7 screening in microbiology diagnostic settings. Similarly, other RT-PCR-based assays have been developed by other companies to screen two new worrying VOCs, B.1.351 and P.1, targeting the E484K and N501Y mutations in the S-gene, which are considered markers for these VOCs.

Our group has implemented a two-step procedure to screen VOCs. First, detection of SARS-CoV-2 is performed using routine RT-PCR (Thermo Fisher Taq-Path RT-PCR) on nasopharyngeal specimens. This step allows for identification of case candidates infected by the B.1.1.7 variant, based on the demonstration of S-gene target failure due to the spike 69/70 deletion. Second, cases where S-gene target failure was not identified are further screened using one of the new assays for early detection of B.1.351/P.1 VOCs (it also includes the detection of the B.1.525 variant of interest [VOI] [SARS-CoV-2 variants ELITe MGB kit]). Finally, B.1.351/P.1 and B.1.525 candidates are confirmed by WGS. For WGS, RNA was reverse-transcribed using random hexamers. Whole-genome amplification of the coronavirus was done with the Artic_nCov-2019_V3 panel of primers (artic.network/ncov-2019). Libraries were prepared using the Nextera Flex DNA library preparation kit (Illumina, Inc., California, USA) and sequenced on the Miseq system (Illumina, Inc.) using the v2 kit.

The purpose of this study is to assess the concordance between RT-PCR-based preassignment of P.1/B.1.351 or B.1.525 candidates and final confirmation by WGS.

A total of 142 COVID-19 cases were candidates possibly infected by non-B.1.1.7 variants, identified by our two-step RT-PCR-based approach from 16 April to 1 June 2021. Of these candidates, 121 (85.21%) harbored the E484K and N501Y mutations (B.1.351/P1 candidates as per commercial kit indications), 12 (8.45%) harbored the E484K mutation (B.1.525 candidates), and none of the previous mutations were found in the remaining nine (6.34%) cases (Table 1).

**TABLE 1:** Assignment of SARS-CoV-2 VOCs based on RT-PCR and WGS from 16 April to 1 June 2021^*a*^

Sample code	Date (day/mo/yr)	*C_T_* value^*b*^	RT-PCR assignment	WGS assignment	Traveler	Country of origin	GISAID code
1	4/16/21	18	Non-P.1/B.1.351/B.1.525	B.1.575.1	No		EPI_ISL_1914373
2	4/16/21	22	P.1/B.1.351	P.1	No		EPI_ISL_1914365
3	4/16/21	23	P.1/B.1.351	P.1	No		EPI_ISL_1914366
4	4/16/21	27	P.1/B.1.351	P.1	No		EPI_ISL_1914367
5	4/17/21	28	Non-P.1/B.1.351/B.1.525	B.1.177	No		EPI_ISL_1914334
6	4/17/21	18	B.1.525	B.1.526	No		EPI_ISL_1914376
7	4/17/21	16	B.1.525	B.1.526	No		EPI_ISL_1914377
8	4/17/21	22	P.1/B.1.351	P.1	No		EPI_ISL_1914368
9	4/17/21	30	Non-P.1/B.1.351/B.1.525	B.1.177.50	No		EPI_ISL_1914335
10	4/19/21	27	P.1/B.1.351	P.1	No		EPI_ISL_3505904
11	4/19/21	30	P.1/B.1.351	P.1	No		EPI_ISL_3505905
12	4/19/21	24	P.1/B.1.351	P.1	Yes	Mexico	EPI_ISL_3505906
13	4/20/21	19	P.1/B.1.351	P.1	No		EPI_ISL_3505907
14	4/20/21	29	P.1/B.1.351	P.1	No		EPI_ISL_3505908
15	4/21/21	21	P.1/B.1.351	P.1	No		EPI_ISL_3505909
16	4/22/21	16	P.1/B.1.351	B.1.621	Yes	Colombia	EPI_ISL_3505910
17	4/22/21	28	P.1/B.1.351	P.1	No		EPI_ISL_3505911
18	4/23/21	17	P.1/B.1.351	P.1	No		EPI_ISL_1914327
19	4/23/21	17	P.1/B.1.351	P.1	No		EPI_ISL_3505912
20	4/23/21	22	P.1/B.1.351	P.1	No		EPI_ISL_3505913
21	4/23/21	19	P.1/B.1.351	B.1.351	No		EPI_ISL_1914328
22	4/23/21	18	P.1/B.1.351	P.1	No		EPI_ISL_3505914
23	4/24/21	20	P.1/B.1.351	P.1	No		EPI_ISL_3505915
24	4/26/21	21	P.1/B.1.351	B.1.621	Yes	Colombia	EPI_ISL_3505916
25	4/27/21	23	P.1/B.1.351	B.1.621	Yes	Colombia	EPI_ISL_1964218
26	4/27/21	20	P.1/B.1.351	P.1	No		EPI_ISL_1964180
27	4/27/21	22	P.1/B.1.351	P.1	Yes	Colombia	EPI_ISL_3505917
28	4/27/21	17	P.1/B.1.351	P.1	Yes	Colombia	EPI_ISL_1964215
29	4/27/21	21	B.1.525	B.1.526	Yes	Dominican Republic	EPI_ISL_3505918
30	4/28/21	29	Non-P.1/B.1.351/B.1.525	B.1.177	No		EPI_ISL_3505919
31	4/28/21	18	P.1/B.1.351	B.1.111	Yes	Colombia	EPI_ISL_3505920
32	4/29/21	17	P.1/B.1.351	P.1	Yes	Colombia	EPI_ISL_3505921
33	4/29/21	29	P.1/B.1.351	P.1	No		EPI_ISL_3505922
34	4/29/21	23	P.1/B.1.351	P.1	No		EPI_ISL_3505923
35	4/29/21	25	P.1/B.1.351	B.1.621	Yes	Colombia	EPI_ISL_3505924
36	4/29/21	24	P.1/B.1.351	B.1.621	Yes	Colombia	EPI_ISL_3505925
37	4/29/21	21	P.1/B.1.351	B.1.621	Yes	Colombia	EPI_ISL_3505926
38	4/29/21	27	P.1/B.1.351	P.1	No		EPI_ISL_3505927
39	4/30/21	18	P.1/B.1.351	P.1	No		EPI_ISL_1964216
40	4/30/21	17	P.1/B.1.351	B.1.351	No		EPI_ISL_3505928
41	4/30/21	13	P.1/B.1.351	P.1	No		EPI_ISL_3505929
42	5/1/21	20	P.1/B.1.351	B.1.351	No		EPI_ISL_3505930
43	5/1/21	23	P.1/B.1.351	B.1.621	Yes	Colombia	EPI_ISL_3505931
44	5/2/21	18	P.1/B.1.351	P.1	Yes	Brazil	EPI_ISL_3505932
45	5/2/21	18	P.1/B.1.351	B.1.621	Yes	Colombia	EPI_ISL_3505933
46	5/3/21	22	P.1/B.1.351	B.1.351	No		EPI_ISL_3505934
47	5/6/21	24	P.1/B.1.351	B.1.351	No		EPI_ISL_2080926
48	5/6/21	13	P.1/B.1.351	B.1.351	No		EPI_ISL_2080927
49	5/6/21	23	P.1/B.1.351	P.1	No		EPI_ISL_3505935
50	5/6/21	25	P.1/B.1.351	B.1.351	No		EPI_ISL_2080930
51	5/6/21	26	P.1/B.1.351	B.1.621	Yes	Colombia	EPI_ISL_2080928
52	5/6/21	28	P.1/B.1.351	P.1	No		EPI_ISL_2080923
53	5/7/21	29	P.1/B.1.351	P.1	No		EPI_ISL_2080886
54	5/7/21	14	P.1/B.1.351	B.1.351	No		EPI_ISL_3505936
55	5/7/21	21	P.1/B.1.351	P.1	No		EPI_ISL_3505937
56	5/7/21	17	P.1/B.1.351	P.1	No		EPI_ISL_2080922
57	5/7/21	30	B.1.525	B.1.280 (E484Q)	No		EPI_ISL_2080885
58	5/8/21	18	P.1/B.1.351	B.1.621	Yes	Colombia	EPI_ISL_3505938
59	5/9/21	20	P.1/B.1.351	B.1.351	No		EPI_ISL_3505939
60	5/10/21	21	B.1.525	B.1.280 (E484Q)	Yes	Dominican Republic	EPI_ISL_2172424
61	5/11/21	16	P.1/B.1.351	P.1	Yes	Colombia	EPI_ISL_3505940
62	5/12/21	17	P.1/B.1.351	B.1.621	Yes	Colombia	EPI_ISL_3505941
63	5/12/21	28	P.1/B.1.351	P.1	No		EPI_ISL_3505942
64	5/12/21	14	P.1/B.1.351	P.1	No		EPI_ISL_3505943
65	5/13/21	19	P.1/B.1.351	B.1.621	Yes	Colombia	EPI_ISL_3505944
66	5/13/21	14	P.1/B.1.351	B.1.351	Yes	Cuba	EPI_ISL_3505945
67	5/13/21	29	P.1/B.1.351	B.1.621	Yes	Dominican Republic	EPI_ISL_3505946
68	5/14/21	20	B.1.525	B.1.1.318	Yes	Senegal	EPI_ISL_3505947
69	5/14/21	30	P.1/B.1.351	P.1	No		EPI_ISL_2178828
70	5/14/21	16	P.1/B.1.351	P.1	Yes	Dominican Republic	EPI_ISL_3505948
71	5/14/21	17	P.1/B.1.351	P.1	Yes	Paraguay	EPI_ISL_3505949
72	5/14/21	17	P.1/B.1.351	B.1.621	Yes	Colombia	EPI_ISL_3505950
73	5/14/21	19	P.1/B.1.351	B.1.621	Yes	Colombia	EPI_ISL_3505951
74	5/14/21	17	Non-P.1/B.1.351/B.1.525	C.37	Yes	Colombia	EPI_ISL_2178831
75	5/14/21	17	P.1/B.1.351	B.1.621	Yes	Colombia	EPI_ISL_2178834
76	5/14/21	30	P.1/B.1.351	P.1	No		EPI_ISL_3505952
77	5/15/21	24	P.1/B.1.351	P.1	Yes	Portugal	EPI_ISL_3505953
78	5/15/21	15	P.1/B.1.351	B.1.621	Yes	Colombia	EPI_ISL_3505954
79	5/16/21	27	P.1/B.1.351	B.1.621	Yes	Colombia	EPI_ISL_3505955
80	5/16/21	17	P.1/B.1.351	B.1.621	Yes	Colombia	EPI_ISL_3505956
81	5/16/21	17	P.1/B.1.351	P.1	Yes	Colombia	EPI_ISL_3505957
82	5/16/21	22	P.1/B.1.351	P.1	No		EPI_ISL_3505958
83	5/17/21	24	P.1/B.1.351	B.1.621	Yes	Colombia	EPI_ISL_3505959
84	5/17/21	30	P.1/B.1.351	P.1	Yes	Unknown	EPI_ISL_3505960
85	5/18/21	15	P.1/B.1.351	P.1	No		EPI_ISL_3505961
86	5/19/21	22	P.1/B.1.351	P.1	No		EPI_ISL_3505962
87	5/19/21	15	P.1/B.1.351	P.1	No		EPI_ISL_3505963
88	5/19/21	17	P.1/B.1.351	B.1.351	Yes	Cuba	EPI_ISL_3505964
89	5/19/21	18	P.1/B.1.351	B.1.623	Yes	Dominican Republic	EPI_ISL_3505965
90	5/19/21	18	P.1/B.1.351	B.1.623	Yes	Dominican Republic	EPI_ISL_3505966
91	5/20/21	26	Non-P.1/B.1.351/B.1.525	B.1	No		EPI_ISL_2304188
92	5/20/21	28	P.1/B.1.351	P.1	No		EPI_ISL_3505967
93	5/20/21	14	P.1/B.1.351	B.1.621	Yes	Colombia	EPI_ISL_3505968
94	5/20/21	31	P.1/B.1.351	P.1	No		EPI_ISL_3505969
95	5/20/21	18	B.1.525	B.1 (E484Q)	Yes	Dominican Republic	EPI_ISL_3505970
96	5/20/21	22	P.1/B.1.351	B.1.351	No		EPI_ISL_3505971
97	5/21/21	27	P.1/B.1.351	B.1.623	No		EPI_ISL_3505972
98	5/21/21	18	B.1.525	B.1.526	No		EPI_ISL_3505973
99	5/21/21	22	B.1.525	B.1.526	No		EPI_ISL_2304191
100	5/21/21	18	P.1/B.1.351	B.1.621	No		EPI_ISL_3505974
101	5/21/21	30	B.1.525	B.1.526	No		EPI_ISL_2304190
102	5/21/21	30	Non-P.1/B.1.351/B.1.525	B.1.617.2	Yes	India	EPI_ISL_2296706
103	5/21/21	21	P.1/B.1.351	P.1	No		EPI_ISL_2304186
104	5/22/21	31	P.1/B.1.351	P.1	No		EPI_ISL_3505976
105	5/22/21	25	P.1/B.1.351	P.1	No		EPI_ISL_3505975
106	5/22/21	17	B.1.525	B.1 (E484Q)	No		EPI_ISL_2304153
107	5/22/21	20	P.1/B.1.351	B.1.623	No		EPI_ISL_3505977
108	5/22/21	18	P.1/B.1.351	P.1	No		EPI_ISL_3505978
109	5/22/21	16	P.1/B.1.351	B.1.621	No		EPI_ISL_3505979
110	5/22/21	24	P.1/B.1.351	P.1	Yes	Colombia	EPI_ISL_2304183
111	5/23/21	22	P.1/B.1.351	B.1.621	Yes	Colombia	EPI_ISL_3505980
112	5/23/21	18	P.1/B.1.351	B.1.621	Yes	Colombia	EPI_ISL_3505981
113	5/24/21	20	P.1/B.1.351	P.1	Yes	Brazil	EPI_ISL_3505984
114	5/24/21	14	P.1/B.1.351	P.1	Yes	Brazil	EPI_ISL_3505983
115	5/24/21	23	P.1/B.1.351	B.1.621	Yes	Colombia	EPI_ISL_3505982
116	5/25/21	26	P.1/B.1.351	P.1	No		EPI_ISL_2402499
117	5/25/21	16	P.1/B.1.351	B.1.351	No		EPI_ISL_3505985
118	5/25/21	14	P.1/B.1.351	B.1.623	No		EPI_ISL_2402506
119	5/26/21	18	P.1/B.1.351	B.1.623	No		EPI_ISL_3505986
120	5/26/21	32	P.1/B.1.351	B.1.1	No		EPI_ISL_3505987
121	5/26/21	25	P.1/B.1.351	P.1	No		EPI_ISL_3505988
122	5/27/21	20	Non-P.1/B.1.351/B.1.525	B.1.617.2	No		EPI_ISL_2402478
123	5/27/21	17	P.1/B.1.351	B.1.621	Yes	Colombia	EPI_ISL_2402508
124	5/27/21	18	P.1/B.1.351	B.1.621	Yes	Colombia	EPI_ISL_2402505
125	5/28/21	16	Non-P.1/B.1.351/B.1.525	B.1.617.2	No		EPI_ISL_2402477
126	5/28/21	20	P.1/B.1.351	B.1.623	No		EPI_ISL_2402512
127	5/28/21	14	P.1/B.1.351	B.1.621	Yes	Colombia	EPI_ISL_3505989
128	5/28/21	16	P.1/B.1.351	B.1.621	Yes	Colombia	EPI_ISL_3505990
129	5/29/21	15	P.1/B.1.351	P.1	Yes	Venezuela	EPI_ISL_2402527
130	5/29/21	22	P.1/B.1.351	B.1.621	Yes	Colombia	EPI_ISL_2402528
131	5/29/21	29	P.1/B.1.351	B.1.351	No		EPI_ISL_2402513
132	5/30/21	17	P.1/B.1.351	B.1.623	Yes	Colombia	EPI_ISL_3505991
133	5/30/21	21	P.1/B.1.351	B.1.621	Yes	Colombia	EPI_ISL_3505992
134	5/30/21	22	P.1/B.1.351	B.1.621	Yes	Colombia	EPI_ISL_3505993
135	5/30/21	23	P.1/B.1.351	B.1.621	Yes	Colombia	EPI_ISL_3505994
136	5/30/21	19	P.1/B.1.351	B.1.621	Yes	Colombia	EPI_ISL_3505995
137	5/30/21	26	P.1/B.1.351	B.1.621	Yes	Colombia	EPI_ISL_3505996
138	5/30/21	19	P.1/B.1.351	B.1.623	Yes	Dominican Republic	EPI_ISL_3505997
139	5/31/21	22	B.1.525	B.1 (E484Q)	No		EPI_ISL_3505998
140	5/31/21	22	P.1/B.1.351	B.1.621	No		EPI_ISL_3505999
141	5/31/21	17	P.1/B.1.351	B.1.621	No		EPI_ISL_3506000
142	6/1/21	24	P.1/B.1.351	B.1.621	No		EPI_ISL_3506001

a Shading indicates discrepancies between RT-PCR and WGS variant assignations.

b *C_T_*, threshold cycle.

WGS of all cases confirmed the RT-PCR-based preassignment in 71 of the cases (58.68%; 57 P.1 [80.28%] and 14 B.1.351 [19.72%]) from the 121 P.1/B.1.351 candidates (Table 1). The remaining 50 cases (41.32%) were wrongly assigned, corresponding to B.1.621 (78%) (39 cases), B.1.111 (2%) (1 case), B.1.623 (18%) (9 cases) and B.1.1 (2%) (1 case) (Table 1).

B.1.621 is a VOI, as defined by the World Health Organization (4). It harbors several mutations affecting the spike protein, including E484K and N501Y (5). There are limited data on the global distribution of this variant, which has become predominant in some regions of Colombia, with up to 7% of sequenced cases. In our series, 33 (84.62%) corresponded to travelers from Colombia, 1 (2.56%) from the Dominican Republic, and 5 (12.82%) were unlinked cases.

None of the B.1.525 preassigned cases was confirmed by WGS (Table 1). All of them were wrongly assigned, corresponding to B.1.526 (50%) (6 cases), B.1.1.318 (8.33%) (1 case), B.1 (25%) (3 cases), and B.1.280 (16.67%) (2 cases). In five cases (41.67%), the E484K mutation was wrongly assigned by RT-PCR, corresponding to E484Q (B.1 and B.1.280 variants; Table 1).

For the remaining cases, for which the RT-PCR-based prescreening did not detect the E484K/N501Y mutations, three (33.33%) corresponded to B.1.617.2, a VOC. In the remaining six cases, different non-VOC/VOIs were identified by WGS, specifically, B.1.575.1, B.1.177, B.1.177.50, C.37, and B.1 (Table 1).

Our findings indicate that incorrect B.1.351/P.1 assignment may occur when using a targeted RT-PCR prescreening approach, based on the identification of a limited number of marker single nuclear polymorphisms (SNPs). The emergent presence of the B.1.621 VOI, sharing the two E484K/N501Y mutations with P.1/B.1.351, is mainly responsible for the incorrect assignments in our study. The misassignments found in our study are not associated with specific technical limitations of the test applied; they are the result of assuming that a proper assignment of VOCs can be performed by targeting a low number of marker mutations. Thus, caution is recommended when applying targeted RT-PCR for VOC screening. In the current context of constant emergence of new variants, commercial solutions devoted to prescreen VOCs should rely on a wider set of mutations to ensure specificity. Mutations targeted by these approaches should be constantly evaluated considering updated WGS data on circulating variants.

## GENOMIC METHODS

We used 11 μl of RNA as the template for reverse transcription using Invitrogen SuperScript IV reverse transcriptase (Thermo Fisher Scientific, MA, USA) and random hexamers (Thermo Fisher Scientific). Whole-genome amplification of the coronavirus was done with an Artic_nCov-2019_V3 panel of primers (Integrated DNA Technologies, Inc., Coralville, IA, USA) (artic.network/ncov-2019) and the Q5 Hot Start DNA polymerase (New England Biolabs, Ipswich, MA, USA). Libraries were prepared using the Nextera Flex DNA library preparation kit (Illumina, Inc., CA, USA) following the manufacturer’s instructions.

Libraries were quantified with the Quantus fluorometer (Promega, WI, USA) before being pooled at equimolar concentrations (4 nM). Next, they were sequenced in pools of up to 17 libraries on the MiSeq system (Illumina, Inc., CA, USA) and the MiSeq reagent microkit v2 (2 × 151 bp) or in pools of up to 96 libraries with the MiSeq reagent (2 × 201 bp).

FastA files above the GISAID thresholds were deposited at GISAID (Table 1). An in-house analysis pipeline was applied to analyze the sequencing reads. The pipeline can be accessed at https://github.com/pedroscampoy/covid_multianalysis. Briefly, the pipeline goes through the following steps: (i) removal of human reads with Kraken (6), (ii) preprocessing and quality assessment of FASTQ files using fastp v0.20.1 (7) (arguments: –cut tail, –cut-window-size, –cut-mean-quality, -max_len1,-max_len2) and fastQC v0.11.9 (8), (iii) mapping with the Burrows-Wheeler Aligner (BWA) v0.7.17 and variant calling using iVAR v1.2.3 (9) using the Wuhan-1 strain sequence (GenBank accession number NC_045512.2) as the reference, and (iv) recalibration of punctual low-coverage positions using joint variant calling. When necessary, informative noncovered positions were analyzed by standard Sanger sequencing with the corresponding flanking primers from the ARTIC v3 set.
